# Comparison of adrenalectomy with conservative treatment on mild autonomous cortisol secretion: a systematic review and meta-analysis

**DOI:** 10.3389/fendo.2024.1374711

**Published:** 2024-05-13

**Authors:** Mohamad Mehdi Khadembashiri, Shahrzad Mohseni, Hamid Harandi, Mahnaz Pejman Sani, Mohamad Amin Khadembashiri, Rasha Atlasi, SeyedAhmad SeyedAlinaghi, Mohammadreza Mohajeri- Tehrani, Bagher Larijani

**Affiliations:** ^1^ Endocrinology and Metabolism Research Center, Endocrinology and Metabolism Clinical Sciences Institute, Tehran University of Medical Sciences, Tehran, Iran; ^2^ Research Center for Antibiotic Stewardship and Antimicrobial Resistance, Imam Khomeini Hospital Complex, Tehran University of Medical Sciences, Tehran, Iran; ^3^ Non-Communicable Diseases Research Center, Endocrine Population Sciences Institute, Endocrinology and Metabolism Research Institute, Tehran University of Medical Sciences, Tehran, Iran; ^4^ Iranian Research Center for HIV/AIDS, Iranian Institute for Reduction of High-Risk Behaviors, Tehran University of Medical Sciences, Tehran, Iran

**Keywords:** mild autonomous cortisol secretion, subclinical Cushing’s syndrome, Cushing’s syndrome, adrenalectomy, conservative, systematic review, meta-analysis

## Abstract

**Objective:**

This systematic review and meta-analysis was conducted to compare the benefits of adrenalectomy and conservative treatment for comorbidities associated with mild autonomous cortisol secretion (MACS) in patients diagnosed with MACS.

**Background:**

MACS is the most common benign hormone-secreting functional adrenal incidentaloma. Overproduction of cortisol is observed in MACS patients, resulting in a variety of long-term health issues, including arterial hypertension (HTN), diabetes mellitus (DM), dyslipidemia, obesity, and osteoporosis; however, the classic clinical manifestations of Cushing’s syndrome (CS) are not present.

**Methods:**

A systematic search was conducted using MEDLINE, Embase, Web of Sciences, and Scopus databases on December, 2023. Two reviewers independently extracted data and assessed the quality of the included articles. A meta-analysis was performed to compare the beneficial effects of adrenalectomy versus conservative management for MACS-related comorbidities.

**Results:**

Fifteen articles were included in this study, which evaluated 933 MACS patients (384 Adrenalectomy and 501 Conservative treatment, and 48 excluded due to incomplete follow-up duration). MACS diagnosis criteria were different among the included articles. All studies, however, stated that there must be no overt CS symptoms. Meta-analysis demonstrates the overall advantage of adrenalectomy over conservative treatment for MACS-related comorbidities (Cohen’s d = -0.49, 95% CI [-0.64, -0.34], p = 0.00). Subgroup analysis indicated that the systolic blood pressure (pooled effect size = -0.81, 95% CI [-1.19, -0.42], p = 0.03), diastolic blood pressure (pooled effect size = -0.63, 95% CI [-1.05, -0.21], p = 0.01), and BMD (pooled effect size = -0.40, 95% CI [-0.73, -0.07], p = 0.02) were significantly in favor of adrenalectomy group rather than conservative treatment but no significant differences between the two treatment groups in other MACS-related comorbidities were reported.

**Conclusion:**

Despite the limited and diverse data, this study demonstrates the advantage of adrenalectomy over conservative treatment for MACS-related comorbidities.

## Graphical Abstract

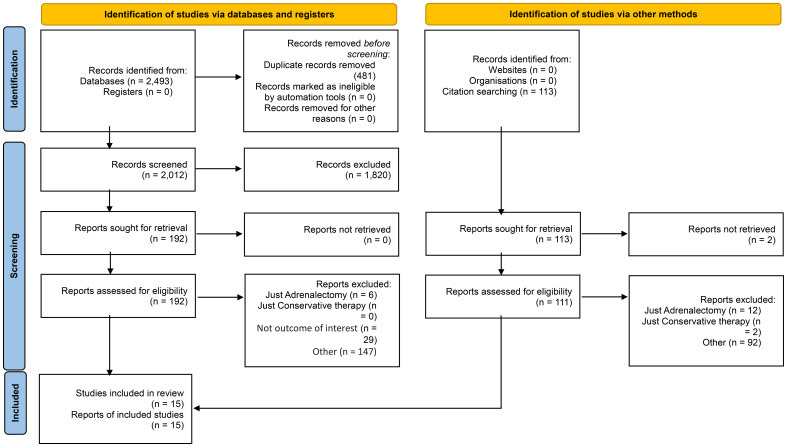

## 1 Introduction

Mild Autonomous Cortisol Secretion (MACS) is initially identified as subclinical Cushing’s syndrome (SCS); however, the term “Subclinical or Preclinical” may not be appropriate for this condition as it implies a transition to Cushing’s syndrome, which is an uncommon occurrence (1–4). In this context, we employ the term “mild autonomous cortisol secretion” as recommended by the European Society of Endocrinology and European Network for the Study of Adrenal Tumors (ESE-ENSAT) (5). MACS is an adrenal incidentaloma, which refers to the incidental findings of an adrenal mass during diagnostic investigations conducted for reasons unrelated to suspected adrenal pathology (6, 7), characterized by increased cortisol production, which is independent to hypothalamic–pituitary–adrenal (HPA) axis, without clinical signs of overt Cushing’s syndrome (5, 8). Over the past decade, this issue has been debated, primarily because of an unclear definition and contentious treatment approaches (9, 10).

The prevalence of adrenal incidentaloma is between 0.4% to 7% And can be and up to 10% in patients > 80 years (7, 11–13), which can be hormone-secreting, nonfunctional, malignant, or benign (14). As hormone overproduction is one of the major clinical concerns in adrenal incidentaloma, MACS should be evaluated precisely since it is recognized as the most common benign hormone-secreting functional adrenal incidentaloma (15). The prevalence of MACS is estimated to be 79 cases per 100,000 people (16), accounting for 5 to 20% of adrenal incidentaloma masses (17, 18).

There is convincing evidence to suggest that MACS can cause physiological effects associated with excessive cortisol levels. These effects encompass a range of long-term health complications, such as arterial hypertension (HTN), diabetes mellitus (DM), dyslipidemia, obesity, and osteoporosis (19–21). Furthermore, there have been documented reports of elevated mortality linked to cardiovascular events in individuals diagnosed with MACS (22–25). The definite treatment for overt Cushing’s syndrome is adrenalectomy, but the optimal treatment for MACS is still controversial (26, 27). Several studies have documented a notable enhancement in comorbidities associated with MACS subsequent to adrenalectomy, while others have not observed any significant alterations in this regard (28–30).

This systematic review and meta-analysis were conducted to enhance comprehension of the available data regarding the positive impacts of adrenalectomy on cardiovascular risk factors and other comorbidities in patients with MACS. To reach this objective, we compared the effect of adrenalectomy with conservative treatment on comorbidities associated with MACS.

## 2 Methods

The research protocol for this study underwent examination and approval by the ethics committee at the Endocrinology and Metabolism Research Institute of Tehran University of Medical Sciences (Ethical code: IR.TUMS.EMRI.REC.1402.013).

### 2.1 Data source and search strategy

The current review is designed based on Preferred Reporting Items for Systematic Reviews and Meta-Analyses (PRISMA) guidelines (31). A systematic search was conducted in several databases using a combination of keywords and MESH terms, including “Cushing Syndrome” or “adrenal incidentalomas’’ or “adrenal mass,” “subclinical Cushing’s syndrome’’ or “preclinical Cushing’s syndrome’’ or “autonomous cortisol secretion’’ or ‘‘Hypercortisolism’’ in combination with “Conservative Treatment”, and “Surgical Procedures, Operative” or “Adrenalectomy” or “General Surgery”. The Search strategy is provided in the Appendix 1. We searched MEDLINE, Embase, Web of Sciences, and Scopus databases in any language on December, 2023. There were no limitations placed on the search based on language and date. An experienced librarian and an endocrinologist with specialized knowledge in the field conducted the planning and implementation of the search strategy. Furthermore, the accuracy of the search strategy has been assessed by other authors.

### 2.2 Inclusion and exclusion criteria

The selected studies were required to meet certain criteria to be considered for inclusion in this study. Specifically, they were required to provide data on both adrenalectomy and conservative treatment in patients diagnosed with MACS. Additionally, the studies were expected to examine at least one of the following outcomes: arterial hypertension, diabetes, dyslipidemia, obesity, osteoporosis, and vertebral fractures.

The excluded studies from the analysis were those that solely presented preoperative data or focused only on adrenalectomy or conservative therapy. Case reports and case series including fewer than ten patients were also excluded, as they were considered reviews and letters. Another exclusion criteria were those studies that encompass patients diagnosed with clinically evident Cushing’s syndrome and other adrenal disorders, including primary hyperaldosteronism, phaeochromocytoma, and non-functional adrenal tumors (NFA). Publications that did not have biochemically confirmed MACS and studies that did not distinguish between clinically evident CS and MACS were also excluded from our review.

In order to request further information or have a complete data, the corresponding authors were contacted via email. Once the desired data were obtained, they were used for analysis. Two researchers, working separately, evaluated the articles’ title, abstract, and full text from the initial search outcomes to select papers that aligned with the inclusion and exclusion criteria.

### 2.3 Data extraction

The data was extracted independently from included publications by two researchers using a standardized piloted web-based form and then were compared. Using full texts and debates, the third researcher made a decision concerning the conflicting and inconsistent data. A total of 13 publications were reviewed in the original language without transcription in English and two in Chinese.

### 2.4 Quality assessment

The risk of bias was evaluated using the Newcastle-Ottawa Scale (NOS) for assessing the quality of non-randomized studies. This tool evaluates how well the sample represented the population of interest, how the comparative group was chosen, how the outcome was assessed, and the length and sufficiency of follow-up (32). The Downs and Black checklist was used for a single study with a randomized controlled trial design (33). The methodological quality of the publications was individually appraised to determine their eligibility for inclusion in a meta-analysis.

### 2.5 Meta-analysis

Using the random-effects model, we conducted a meta-analysis to pool estimates from included studies. To account for heterogeneity between studies and within-study variability, a random-effects model was used instead of a fixed-effects model. The I^2^ statistic was used to calculate the percentage of total between-study variation owing to heterogeneity rather than chance ranging from 0 to 100%. Low, moderate, and high heterogeneity are represented by I^2^ values of 25, 50, and 75%, respectively. STATA version 17 was used for data analysis and statistical procedures. (StataCorp. 2021. Stata Statistical Software: Release 17. College Station, TX: StataCorp LLC). For negative variables, the Cohen’s d value was used to calculate the effect size of the difference between the means of the two adrenalectomy and conservative treatment groups in terms of standard deviations. The Hedges’s g effect size was used for other variables including high-density lipoprotein (HDL) and bone mineral density (BMD). A significance threshold of P<0.05 was considered to determine statistical significance.

We depicted the forest plot, which illustrates the individual effect sizes and their corresponding confidence intervals for each study included in the analysis. We produced the Galbraith plot to demonstrate heterogeneity across studies. Furthermore, the funnel plots in Appendix shows a graphical representation of publication bias.

### 2.6 Publication bias assessment

Publication bias was evaluated through the utilization of funnel plots, while the extent of asymmetry was examined using Egger’s regression test (34). The analysis was performed in Stata version 17 using the “meta” package.

## 3 Results

### 3.1 Characteristics and overview of studies

Our search yields a total of 2606 articles (2493 studies via databases and 113 via citation searching) that underwent title/abstract screening, eventually 15 references included in this study (28, 35–48), including 13 retrospective cohort and two prospective cohort studies. Six of the studies were from Asian centers (3 Japan and 3 China) and nine were from Europe centers (mostly from Italy). All of the included studies evaluated the comparative effects of conservative treatment and adrenalectomy for the MACS patients. Other studies which assess only one of these treatments were excluded. The characteristics of included articles are shown in **Table 1**. A total of 933 patients with MACS were included in this systematic review, among which 885 patients had completed the follow-up. A total of 384 patients underwent surgery (adrenalectomy) with a mean age of 56.1 years (Males: 30.7%, one study didn’t identify gender) (mean duration of follow-up: 34.8 months, one study only reported the median duration of follow-up) and 501 conservatively managed with the mean age of 60.4 years (Males: 40.4%, one study didn’t identify gender) (mean duration of follow up: 37.6 months, one study only reported median duration of follow-up). The average tumor size was 31.7 mm and 26.8 mm in the adrenalectomy and conservative groups, respectively. (Three studies didn’t report tumor size). The results show that 62.9% (number of patients: 345 of 548) of individuals with MACS experience hypertension, 29.2% (number of patients: 137 of 249) show impaired glucose metabolism (diabetes or impaired glucose tolerance), 41.4% (number of patients: 162 of 391) have dyslipidemia, and 38.2% (number of patients: 142 of 372) were affected by obesity. There were no reports of progression to overt Cushing’s syndrome, except for one study, which reported a female in a conservative therapy group developing symptoms of overt Cushing’s syndrome during follow-up.

**Table 1:** Characteristics of Included studies.

Author (Year)	Country	Study Type	Study Size(n)	Adrenalectomy				Conservative treatment				
				Sex(n)	Age (Years)	Duration of F/U (months)	Tumor size (mm)	Sex(n)	Age (years)	Duration of F/U (months)	Tumor size (mm)	Overt CS progression(n)
Akaza et al., 2011 (35)	Japan	Cohort Retrospective	16	2 m 6 f	55.9 ± 10.5	28 ± 7	25.5 ± 6.8	2 m 6 f	57.2± 12.5	40 ± 19	27.1 ± 10.1	–
Toniato (2009) (36)	Italy	Randomized Control trial	45	12 m 11 f	63 ± 4·1	92·4 (24–204)	29·8 ± 29	10 m 12 f	64 ± 1·8	92·4 (24–204)	26·5 ± 4·2	0
Chiodini (2010) (28)	Italy	Retrospective	41	5 m 20 f	54·8 ± 11·6	29·4 ± 13·8)	32 ± 1	3 m 13 f	64·4 ± 10·1	36·4 ± 11·7	24 ± 8	–
Iacobone (2012) (37)	Italy	Retrospective	35	12 m 8 f	56·8 ± 11·9	54 ± 34	39·1 ± 6·5	8 m 7 f	57·4 ± 11	56 ± 37	38·5 ± 7·4	0
Tsuiki (2008) (38)	Japan	Retrospective	20	2 m 8 f	58·4 ± 9·8	13·8 ± 3·8	34·5 ± 9·7	4 m 6 f	60·9 ± 11·3	27·3 ± 15·2	28 ± 5·8	1 f
Gurrieri (2010)	Italy	Retrospective	47	4 m 15 f	54·8 ± 2·7	46·6 ± 3·9	–	10 m 18 f	57·8 ± 2·2	50·1 ± 29·2	–	0
Wang (2018) (40)	China	Retrospective	87	18 m 30 f	51.8 ± 10.2	32.5 ± 10.6	30.1 ± 8.4	20 m 19 f	53.2 ± 12.1	30.1 ± 13.1	29.9 ± 10.1	0
Salcuni (2016) (41)	Italy	Retrospective	55	10 m 22 f	61.3 ± 8.1	39.9 ± 20.9	34 ± 12	13 m 10 f	65.4± 7.1	27.7 ± 11.1	28 ± 9	–
Araujo-Castro (2022) (42)	Spain	Retrospective	259	42	59.2 ± 8.67	30.0 ± 44.9	42.5 ± 16.70	217	65.8 ± 10.50	47.4 ± 43.78	24.0 ± 10.35	–
Petramala (2017) (6)	Italy	Retrospective	70	6 m 20 f	58.7 ± 7.12	12 (9–15)	24 ± 14	21 m 23 f	63.9 ± 9.9	12 (9–15)	22.5 ± 9	–
Kawate (2014) (44)	Japan	Retrospective	27	2 m 13 f	55.3 ± 9.4	Median: 63.6	32.9 ± 17.6	6 m 6 f	66.3± 8.8	Median: 63.6	27.8 ± 5.7	–
Rossi (2000) (45)	Italy	Prospecive	12	1 m 4 f	55 ± 8.29	38 (12–63)	–	2 m 5 f	64.8 ± 6.72	28 (9–73)	–	–
Liu (2020) (46)	China	Retrospective	56 (42 complete F/U)	7 m 24 f	51 ± 11	11.6 ± 7.2	–	3 m 8 f	59 ± 18	12.0 (6.0 - 24.0)	–	0
Li (2017) (47)	China	Retrospective	130(96 complete F/U)	19 m 45 f	52.1 ± 9.5	28.2 ± 19.0	28.0 ± 10.7	12 m 20 f	54.0± 8.9	36.1 ± 16.6	23.3 ± 9.2	–
Ricciato (2014) (48)	Italy	Retrospective	33	5 m 11 f	54.7 ± 12.4	30.9 ± 16.1	29 ± 11	5 m 12 f	52.1 ± 15.8	31.5 ± 26.3	23 ± 8	0

F/U, Follow-Up; Overt CS, Overt Cushing’ Syndrome.

### 3.2 Diagnostic criteria and definition of MACS

The diagnostic criteria for MACS varied between studies (see **Table 2**). One study yielded no information on diagnosis. However, all other studies agreed that there are no overt Cushing’s syndrome symptoms in patients with MACS. The cortisol cutoff after 1 mg-overnight-Dexamethasone Suppression Test (DST) varied; 5 studies used 3 μg/dl, 5 studies chose 1.8 μg/dl, and 1 study chose 2.5 μg/dl as a diagnostic criterion for cortisol level after 1mg-DST. The maximal cutoff was 5 μg/dl, which was used in three studies. Other criteria, such as low Adrenocorticotropic Hormone (ACTH) level, elevated urinary free cortisol (UFC), Low dehydroepiandrosterone sulfate (DHEA-S), 8 mg-overnight-DST, and imaging were evaluated in some of the included studies. The definition of comorbidities varied across studies, as were the “improvement” and “deterioration” of each specific comorbidity. (See **Table 3**)

**Table 2:** Definition of mild autonomous cortisol secretion of the included studies.

Author(year)	Cushing Features	Overnight DST, cortisol cutoff (dexamethasone dose)	8mg overnight DST, cortisol cutoff	UFC	ACTH	Other	Imaging
Akaza et al., 2011 (35)	None	3 µg/dL (1mg)	1 µg/dL	–	<10 pg/mL	and at least one of the following: 1. ACTH<10 pg/mL 2. Decreased response to CRH 3.loss of cortisol diurnal rhythm (45mg/dl at midnight)	unilateral radioactive uptake, as determined by adrenal scintigraphy.
Toniato (2009) (36)	None	2.5 µg/dL (1mg)	–	Elevated	Low	DHEA-S – low	–
Chiodini (2010) (28)	None	3 µg/dL (1mg)	–	>70µg/24h	<10 pg/mL	–	–
Iacobone (2012) (37)	None	5 µg/dL (1mg)	–	>76µg/24h	<10 pg/mL		–
Tsuiki (2008) (38)	None	3 µg/dL (1mg)	1µg/dL	–	–	Normal basal cortisol and one of following: 1.low DHEA-S 2.low ACTH 3.loss of circadian cortisol rhythm	unilateral uptake on scintigraph
Gurrieri (2010)	–	–	–	–	–	–	–
Wang (2018) (40)	None	1.8 µg/dL (1mg)	–	>300 µg/24h in two of the three consecutive collections	<10pg/mL	At least one of the following: 1.DHEA-S – low 2.ACTH<10pg/mL 3.UFC>300 µg/24h in two of the three consecutive collections	unilateral renal AI, tumor maximum diameter greater than 1 cm, uniform density, smooth edges, no significant signs of malignancy
Salcuni (2016) (41)	None	5.0 mg/dl (1mg) Or 3.0 mg/dl (1mg)	–	>70µg/24h	<10 pg/ml	5.0 mg/dl (1mg) Or At least 2 of these 3 criteria: 1.DST Cutoff 3.0 mg/dl (1mg) 2.UFC>70µg/24h 3. ACTH<10 pg/ml	CT scan: homogeneous, hypodense and, well-shaped features adrenal masses. unenhanced CT scan
Araujo-Castro (2022) (42)	None	Confirmed: 5µg/dL (1mg) Possible: 1.8 - 5 µg/dL (1mg)	–	–	–	–	Detection of a unilateral adrenal mass (size >1 cm)
Petramala (2017) (6)	None	1.8 μg/dl (1mg)	–	>100 mcg/24h	<10 pg/ml	two or more of the following: 1.High UFC 2.morning cortisol >1.8 μg/dl after 1 mg overnight DST 3.morning ACTH levels suppressed	–
Kawate (2014) (44)	None	1.8 μg/dl (1mg)	–	–	low early-morning plasma ACTH levels,	low serum DHEA-S no diurnal changes in serum cortisol level	Increased uptake on adrenal scintigraphy
Rossi (2000) (45)	None	3 µg/dL (2mg)	–	>2 SD above normal range	low	–	–
Liu (2020) (46)	None	5 µg/dL (1mg)	–	elevated	low or suppressed	–	–
Li (2017) (47)	None	1.8 μg/dl (1mg)	–	–	<2.2 pmol/L	Pathology confirmed Adenoma	–
Ricciato (2014) (48)	None	1.8 μg/dl (1mg)	–	–	<10 pg/ml	At least two of the following: 1.DST(1mg) 2.ACT<10 pg/ml 3.Free urinary cortisol>137 lg/24 h 4.Midnight serum cortisol >50 ng/ml	–

DST, dexamethasone suppression test; UFC, urinary free cortisol; ACTH, Adrenocorticotropic hormone; DHEA-S, dehydroepiandrosterone sulfate.

**Table 3:** Definition of comorbidities associated with MACS.

	Definition of Comorbidities				
Author (Year)	HTN	DM/IGT	Obesity	Dyslipidemia	Other
Akaza et al., 2011 (35)	SBP >140 and/or DBP >90 Or on antihypertensive drugs	IGT: FBS 110–125mg/dl and/or 2hours plasma glucose 140–199mg/dl on the 75-gr oral glucose tolerance test (OGTT) DM: FBS ≥126mg/dl and/or 2hours plasma glucose >200mg/dl on the 75-gr OGTT or elevated HbA1c ≥6.1 or treatment with antidiabetic drugs	Obese: BMI >25 kg/m2	Triglycerides ≥150 mg/dl HDL-C < 40mg/dl LDL-C ≥ 140mg/dl Or on treatment with antidyslipidemic drugs	–
Toniato (2009) (36)	SBP >150 and DBP >90	IFG: fasting glucose >110 mg/dl DM: fasting glucose >126 mg/dl or treatment with antidiabetic drugs	Overweight: BMI 25-30 kg/m2 Obese: BMI >30 kg/m2	Triglycerides >150 mg/dl HDL<40 mg/dl in men HDL<50 mg/dl in women	Osteoporosis was diagnosed by measuring a patient’s BMD, evaluated by T-score dual energy x-ray absorptiometry
Chiodini (2010) (28)	SBP >130 and DBP >80 Or on antihypertensive drugs	WHO criteria (49) or on treatment with antidiabetic drugs	Obese: BMI >30 kg/m2	Triglycerides >150 mg/dl HDL<40 mg/dl in men HDL<50 mg/dl in women Or on treatment with antidyslipidemic drugs	–
Iacobone (2012) (37)	SBP ≥140 DBP ≥90 or on antihypertensive treatment	IGT: FBS >110 mg/dl DM: FBS >126 mg/dl or treatment with antidiabetic drugs	Overweight: BMI 25-29.9 kg/m2 Obese: BMI ≥30 kg/m2	Triglycerides >150 mg/dl HDL<40 mg/dl in men HDL<50 mg/dl in women Or on treatment with antidyslipidemic drugs	osteoporosis’’ was defined by a T-score less than-2.5 SD, whereas ‘‘osteopenia’’ by T-score between -1 and -2.5 SD
Tsuiki (2008) (38)	SBP >140 and/or DBP >90 or on antihypertensive treatment	DM: FBS >126 mg/dl or 2hours plasma glucose >200 mg/dl IGT: FPG 110-125 and/or 2hours plasma glucose 140-199 mg/dl on 75-gr OGTT or treatment with antidiabetic drugs	Obesity: BMI ≥25 kg/m2	Total cholesterol >220 mg/dl Or on treatment with antidyslipidemic drugs	–
Gurrieri (2010)	SBP >150 and DBP >90	IGT: FBS >110 mg/dl DM: FBS >126 mg/dl	Overweight: BMI 27-30 kg/m2 Obese: BMI >30 kg/m2	Triglycerides >150 mg/dl HDL<40 mg/dl in men HDL<50 mg/dl in women	–
Wang (2018) (40)	–	–	–	–	–
Salcuni (2016) (41)	SBP >140 and/or DBP >90 or on antihypertensive treatment	WHO criteria (49)	–		BMD was measured by dual-energy X-ray absorptiometry at lumbar spine (LS, precision 1.0%) and femoral neck (FN, precision 1.8%) (Z-Score) Vertebral fractures were identified through a visual examination utilizing the semiquantitative (SQ) visual assessment method as detailed by Genant and colleagues (50).: According to this approach, fractures observed in lateral thoraco-lumbar spine radiographs were characterized as reductions exceeding 20% in the height of the anterior, middle, or posterior vertebral segments. Upon examination of lateral spine radiographs, vertebrae were categorized as intact (SQ grade 0) or displaying varying levels of deformity, including mild (approximately 20–25% compression), moderate (25–40% compression), and severe (over 40% compression), corresponding to SQ grades 1, 2, and 3, respectively.
Araujo-Castro (2022) (42)	SBP >140 and/or DBP >90 or on antihypertensive treatment	based on current standards (51)	Obese: BMI ≥30 kg/m2	based on current standards	Cardiovascular disease encompasses ischemic heart disease, heart failure, transient ischemic attack, or acute stroke.
Petramala (2017) (6)	–	–	–		Metabolic syndrome defined by ATP III-NCEP criteria (52)
Kawate (2014) (44)	SBP >140 and/or DBP >90 or on antihypertensive treatment	DM: FBS ≥126 mg/dL and/or a random BS ≥200 mg/dL and/or HbA1c ≥6.5% and/or use of antidiabetic agents IGT: FBS ≥110 mg/dL and/or random BS 140–199 mg/dL	Obesity: BMI ≥25 kg/m2	total cholesterol level ≥220 mg/dL and/or LDL ≥140 mg/dL and/or HDL<40 mg/dL and/or TG ≥150 Or on treatment with antidyslipidemic drugs	–
Rossi (2000) (45)	–	–	–	–	–
Liu (2020) (46)	–	–	–	–	–
Li (2017) (47)	SBP >140 and/or DBP >90 or on antihypertensive treatment	Not defined exactly	Not defined exactly	Not defined exactly	–
Ricciato (2014) (48)	SBP ≥130 and/or DBP ≥85	IGT: FBS≥110 mg/dl	Obesity: BMI ≥30 kg/m2	Triglycerides ≥150 mg/dl and/or HDL <40 mg/dl (men) and<50 mg/dl (women)	Waist circumference>102 cm (men) or>88 cm (women)

HTN, Hypertension; DM, Diabetes mellitus; IGT, Impaired Glucose Tolerance; HDL, high-density lipoprotein; LDL, low-density lipoprotein; DBP, diastolic blood pressure; SBP, Systolic blood pressure; BMI, Body Mass Index; OGTT, Oral glucose tolerance test.

### 3.3 Quality assessment

All included studies were observational (retrospective or prospective cohort) except one study with randomized control trial design. The sample size was not representative of most studies. Most studies reported a follow-up duration of more than 30 months, but it is still debatable whether this duration is sufficient to detect a significant change in outcome. Overall, the studies included exhibited a low to very low quality, and there was considerable heterogeneity in the data across the various studies. (See **Table 4**)

**Table 4:** The Newcastle-Ottawa Scale (NOS) for assessing the quality of studies.

Studies		Selection			Comparability		Outcome		Overall Score (out of 8)
Author, year	Representativeness of the exposed cohort	Selection of the non-exposed cohort	Ascertainment of exposure	Demonstration that outcome of interest was not present at start of study	Comparability of cohorts on the basis of the design or analysis	Assessment of outcome	Was follow-up long enough for outcomes to occur	Adequacy of follow up of cohorts	
Akaza et al., 2011 (35)	*		*	*	*	*		*	6
Chiodini (2010) (28)	*		*	*	*	*		*	6
Iacobone et al., 2012 (37)	*		*	*		*	*	*	6
Tsuiki et al., 2008 (38)	*		*	*		*	*	*	6
Gurrieri et al., 2010	*		*	*		*	*	*	6
Wang et al., 2018 (40)	*		*	*		*	*	*	6
Salcuni et al., 2016 (41)	*		*	*	*	*	*	*	7
Araujo-Castro et al., 2022 (42)	*		*	*		*	*	*	6
Petramala et al., 2017 (6)	*		*	*		*		*	5
Kawate et al., 2014 (44)	*		*	*		*	*	*	6
Rossi et al., 2000 (45)	*		*	*		*	*	*	6
Liu et al., 2020 (46)	*		*	*		*		*	5
Li et al., 2017 (47)	*		*	*		*		*	5
Ricciato et al., 2014 (48)	*		*	*		*	*	*	6

### 3.4 Publication bias

The funnel plot exhibited a symmetrical distribution of data points across the funnel, suggesting that the presence of publication bias was improbable. In addition, the Egger regression test showed that there was no significant difference in the degree of asymmetry of the funnel plot (p = 0.43) (**Supplementary Figures 1–6**).

### 3.5 Adrenalectomy versus conservative treatment outcome for MACS-related comorbidities

#### 3.5.1 Meta analysis

Results of the subgroup analysis indicated no significant differences between the two treatment groups in various parameters, including body weight, body mass index (BMI), fasting blood sugar (FBS), glycated hemoglobin (HbA1C), total cholesterol, low-density lipoprotein (LDL) cholesterol, high –density lipoprotein (HDL) cholesterol, and triglyceride (TG). However, systolic blood pressure, diastolic blood pressure, and bone mineral density (BMD) (pooled effect size = -0.40, 95% CI [-0.73, -0.07], p = 0.02) were significantly in favor of adrenalectomy group rather than conservative treatment. The adrenalectomy group demonstrated a significant overall advantage over the conservative treatment group regarding negative variates of mild autonomous cortisol secretion following the procedure (Cohen’s d = -0.49, 95% CI [-0.64, -0.34], p = 0.00). However, substantial heterogeneity was observed (I^2 = 51.94%).

#### 3.5.2 Blood pressure

**Figure 1:**
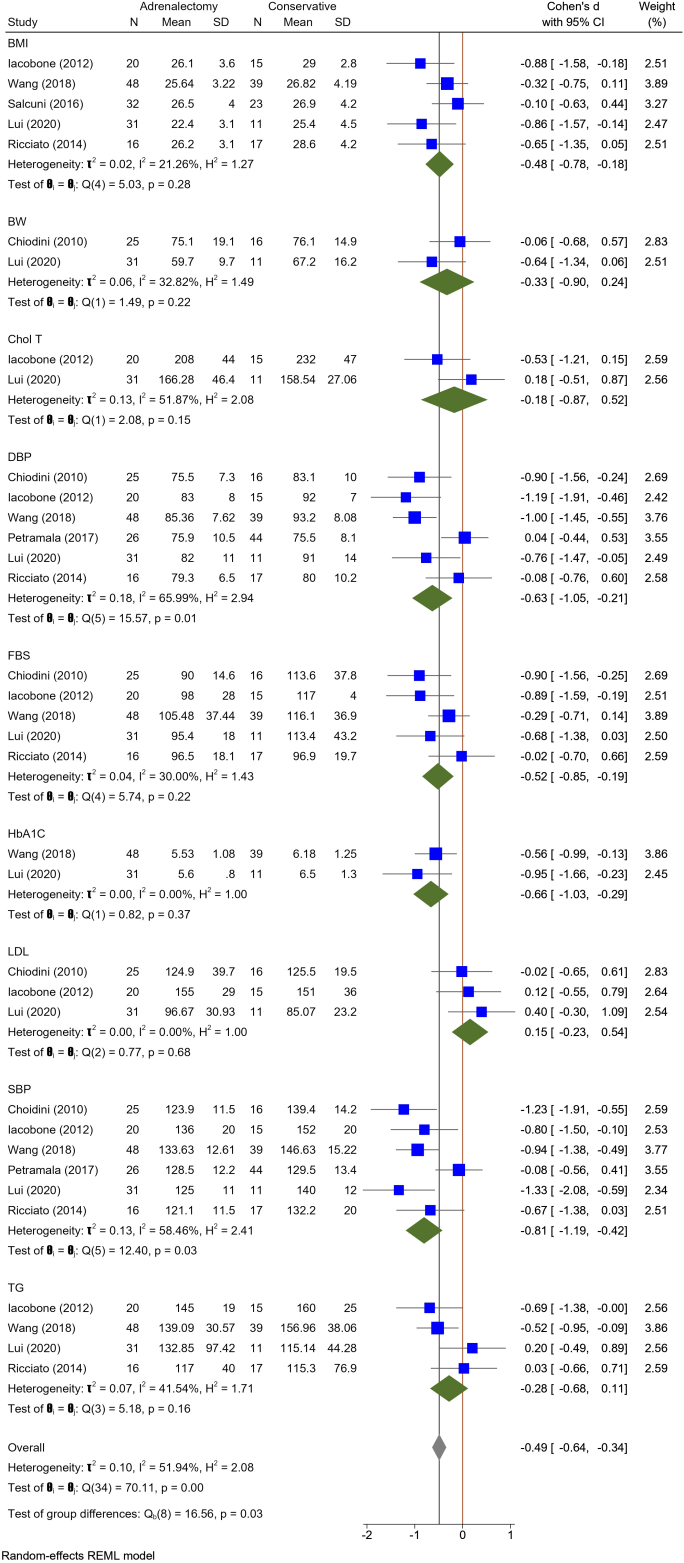
A comparison of the BMI, BW, Total Cholesterol, DBP, FBS, HbA1c, LDL, SBP, and triglyceride levels between the Adrenalectomy and conservative treatment groups. BMI, Body Mass Index; BW, Body Weight; Chol T, Total Cholesterol; DBP, Diastolic Blood Pressure; HbA1c, glycated hemoglobin; LDL, Low Density Lipoprotein; SBP, Systolic Blood Pressure; TG, triglyceride.

Twelve studies evaluated HTN; in the adrenalectomy group, 300 patients with MACS were assessed for HTN, of whom 133 patients that underwent adrenalectomy showed improvement regarding HTN, and only 2 patients exhibited HTN deterioration. In contrast, 248 patients were assessed for HTN in the conservative treatment group; HTN improved in only 3 patients, and 177 patients experienced HTN worsening. Six studies were included in meta-analysis, revealing that the changes in systolic blood pressure (SBP) exhibited a pooled effect size of -0.81 (95% CI [-1.19, -0.42], p = 0.03), and diastolic blood pressure (DBP) demonstrated a pooled effect size of -0.63 (95% CI [-1.05, -0.21], p = 0.01). Heterogeneity analysis revealed moderate heterogeneity for both SBP and DBP among the included studies (SBP: I^2 = 58.46%, H^2 = 2.41, t^2 = 0.13, DBP: Heterogeneity: t2 = 0.18, I2 = 65.99%, H2 = 2.94). These findings support the notion that adrenalectomy is more advantageous than conservative treatment in terms of achieving a significant improvement in both SBP and DBP (**Figure 1**).

#### 3.5.3 Glucose metabolism

In eleven studies, glucose metabolism including DM or Impaired Glucose Tolerance (IGT) was assessed. A total of 260 patients in the adrenalectomy group were evaluated for their glucose metabolism; 61 patients reported an improvement in fasting blood sugar (FBS) levels, while only one patient reported an increase. However, within the conservative treatment group of 209 patients, 3 cases improved, and 42 patients demonstrated FBS-level deterioration. Five Studies included in meta-analysis did not show differences in FBS levels of adrenalectomy group compared to the conservative treatment group with pooled effect size of -0.52 (95%CI [-0.85, -0.19], P = 0.22). Furthermore, the meta-analysis incorporating two studies revealed no substantial distinction between these groups concerning HbA1c levels. The pooled effect size was -0.66 (95% CI [-1.03, -0.29], p = 0.37), indicating a lack of statistically significant differences in HbA1c values between two compared groups (**Figure 1**). Heterogeneity analysis indicated low heterogeneity among the included studies for FBS (I^2 = 30.00%, H^2 = 1.43, t^2 = 0.04) and minimal heterogeneity among the included studies (I^2 = 0.00%, H^2 = 1.00, t^2 = 0.00), indicating a high degree of consistency in the findings across studies regarding HbA1c levels.

#### 3.5.4 Lipid profile

**Figure 2:**
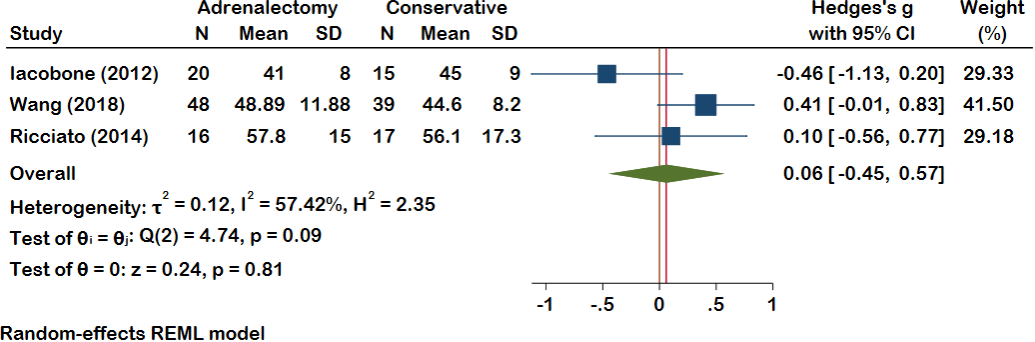
A comparison of the HDL levels between the Adrenalectomy and conservative treatment groups. HDL, High-Density Lipoprotein.

In Ten included studies, Authors evaluate the lipid profile of patients with MACS. Improvement in dyslipidemia was seen in 34 patients in the adrenalectomy group (total patients: 214) and 5 patients in the conservative treatment group (total patients: 177). Dyslipidemia deteriorated in 6 MACS patients who had adrenalectomy and 28 patients who did not. The meta-analysis findings indicate an absence of statistically significant differences between the adrenalectomy group and the conservative group concerning various lipid profile parameters, including HDL (pooled effect size of 0.06 and 95% CI [-0.45, 0.57], P = 0.81), LDL (pooled effect size of 0.15 and 95% CI [-0.23, 0.54], P = 0.68), TG (pooled effect size of -0.28 and 95% CI [-0.68, 0.11], P = 0.16), and total cholesterol (pooled effect size of -0.18 and 95% CI [-0.87, 0.52], P = 0.15) (**Figures 1**, **2**). Nevertheless, certain studies have demonstrated the favorable impact of adrenalectomy on lipid profiles. For instance, Akaza et al., 2011 (35) reported a significantly improvement in the overall lipid profile with adrenalectomy compared to conservative treatment. In the studies conducted by Wang et al. (42) Araujo-Castro et al., adrenalectomy exhibited a significant improvement in TG levels compared to conservative treatment. Furthermore, three studies documented a significant improvement in HDL levels following adrenalectomy compared to the levels observed before the procedure. Heterogeneity results also demonstrated minimal heterogeneity for LDL (t2 = 0.00, I2 = 0.00%, H2 = 1.00), moderate for cholesterol total, TG, and HDL (Chol: t2 = 0.13, I2 = 51.87%, H2 = 2.08; TG: t2 = 0.10, I2 = 51.94%, H2 = 2.08; HDL: t2 = 0.12, I2 = 57.42%, H2 = 2.35).

#### 3.5.5 Weight

Among the included studies, five were considered in the meta-analysis for Body Mass Index (BMI), while two studies were included for Body Weight (BW). Nevertheless, the analysis revealed no significant difference between adrenalectomy and conservative treatment concerning BMI (pooled effect size of -0.48, 95% CI [-0.78, -0.18], p = 0.28) and BW (pooled effect size of -0.33, 95% CI [-0.90, 0.24], p = 0.22) (**Figure 1**). Heterogeneity analysis revealed a relatively low heterogeneity among the included studies (I^2 = 32.82%, H^2 = 1.49, t^2 = 0.06).

#### 3.5.6 Bone

**Figure 3:**
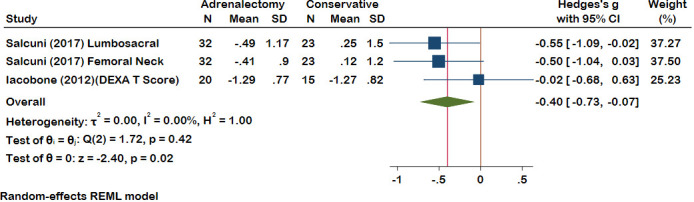
A comparison of the BMD levels between the Adrenalectomy and conservative treatment groups. BMD, Bone Mineral Density.

Within the meta-analysis, bone mineral density (BMD) exhibited a pooled effect size of -0.40 (95% CI [-0.73, -0.07], p = 0.02), decisively favoring the adrenalectomy over conservative treatment (**Figure 3**). Heterogeneity results also demonstrated minimal heterogeneity (I^2 = 00.00%, H^2 = 1.00, t^2 = 0.00). Only one study examined vertebral fractures (VFx), where 32 patients underwent adrenalectomy. Prior to the procedure, 15 of the 32 patients had fractures, but only three new Fx developed following surgery until the end of the follow-up. In another group with 23 conservatively treated patients, 15 of the 23 patients had VFx at the beginning of treatment, whereas, by the end of the follow-up period, 12 patients experienced new VFx. It has been shown that adrenalectomy effectively reduced VFx compared to conservative treatment.

#### 3.5.7 Mental status

One study evaluated mental health using the Short Form 36 Mental Health Component Summary (SF-36 MCS) test and found that adrenalectomy significantly improved mental health (p = 0.003), whereas conservative treatment did not yield similar outcomes.

**Table 5** presents a comprehensive summary of all comorbidities along with the results from each individual study.

**Table 5:** Conclusion of adrenalectomy vs. conservative treatment of each included study.

Author (Year)	Adrenalectomy (n)									Conservative (n)									Conclusion
	Sample size	HTN		DM/IGT		Obesity		Dyslipidemia		Sample size	HTN		DM/IGT		Obesity		Dyslipidemia		
		Before F/U	After F/U	Before F/U	After F/U	Before F/U	After F/U	Before F/U	After F/U		Before F/U	After F/U	Before F/U	After F/U	Before F/U	After F/U	Before F/U	After F/U	
Akaza et al., 2011 (35)	8	5	Imp: 5 Wors: 0	3 DM 3 IGT	Imp: 4 Wors: 0	2	Imp: 4 Wors: 0	3	Imp: 1 Wors: 0	8	4	Imp: 0 Wors: 3	1 DM 1 IGT	Imp: 0 Wors: 0	1	Imp: 1 Wors: 1	5	Imp: 0 Wors: 3	1. Adrenalectomy was effective in improving HTN (P:0.005), DM/IGT(p:0.025), and dyslipidemia (P: 0.044) Compared to conservative treatment. 2. Adrenalectomy was NOT effective in improving BMI compared to conservative treatment. (P:0.085)
Toniato (2009) (36)	23	18	Imp: 12 Wors: 6	8 DM	Imp: 5 Wors: 0	6	Imp: 3 Wors: 0	8	Imp: 3 Wors: 0	22	15	Imp: 0 Wors: 5	6 DM	Imp: 0 Wors: 2	6	Not reported	7	Imp: 0 Wors: 3	1. Adrenalectomy effectively improved HTN after follow-up duration compared to the baseline of the surgical group. (P:0.046)
Chiodini (2010) (28)	25	14	Imp: 14 Wors: 0	7 DM	Imp: 12 Wors: 0	Mean BMI: 29.8 ± 6.1	Imp: 8 Wors: 2	12	Imp: 9 Wors: 6	16	10	Imp: 0 Wors: 8	4 DM	Imp: 0 Wors: 6	Mean BMI: 29.1 ± 4.8	Imp: 2 Wors: 4	5	Imp: 3 Wors: 8	1. Adrenalectomy effectively improved HTN (P<0.001), and FBS (P<0.05), after follow-up duration compared to the baseline of the surgical group. (P<0.001) 2. Conservative therapy leads to increase HTN (P<0.05), and FBS (P<0.05), compared to the baseline of the Conservative group. 3. Adrenalectomy was effective in reducing BW Compared to conservative treatment. (P:0.05) 4. Adrenalectomy Was NOT effective in reducing LDL (P: 0.341) compared to the baseline of the surgical group; however, it significantly protects from worsening LDL levels.
Iacobone (2012) (37)	20	15	Imp: 8 Wors: 0	10 DM or IGT	Imp: 5 Wors: 0	15	Imp: 6 Wors: 0	10	Imp: 2 Wors: 0	15	12	Imp: 0 Wors: 3	6 DM or IGT	Imp: 0 Wors: 2	12	Imp: 0 Wors: 3	7	Imp: 0 Wors: 0	1. Adrenalectomy effectively improved HTN (P:0.002), and FBS (P<0.032) after follow-up duration compared to the baseline of the surgical group. 2. Adrenalectomy Was NOT effective in improving dyslipidemia (P:0.47), and BMI compared to the baseline of the surgical group. 3. Conservative therapy leads to increase HTN (P: 0.05) compared to the baseline of the Conservative group.
Tsuiki (2008) (38)	10	6	Imp: 5 Wors: 0	9	Imp: 2 Wors: 0	3	Imp: 0 Wors: 0	3	Imp: 6 Wors: 0	12	4	Imp: 0 Wors: 2	6	Imp: 0 Wors: 3	3	Imp: 0 Wors: 2	3	Imp: 0 Wors: 2	1. About half of the patients in the conservative group showed a deterioration of cardiovascular risk factors; most of the patients significantly improved the risks after adrenalectomy.
Gurrieri (2010)	19	12	Imp: 9 Wors: 0	Not reported	–	9	average reduction: 2.5 kg/m2 in overweight cases	Not reported	–	28	20	Imp: 0 Wors: 0	Not reported	–	Not reported	–	Not reported	–	1. Adrenalectomy effectively increased HDL and reduced SBP (P<0.005) and DBP (P<0.005), and BMI (P<0.05) after follow-up duration compared to the baseline of the surgical group. 2. Adrenalectomy was NOT effective in reducing FBS, TG, Cholesterol, compared to the baseline of the surgical group. 3. Conservative treatment leads to increase FBS compared to the baseline of the Conservative group.
Wang (2018) (40)	48	Not reported	Imp: 22 Wors: 1	Not reported	Imp: 4 Wors: 0	–	–	Not reported	Imp: 5 Wors: 0	39	Not reported	Imp: 0 Wors: 5	Not reported	Imp: 0 Wors: 1	–	–	Not reported	Imp: 0 Wors: 2	1. Adrenalectomy was effective in improving HTN (P: 0.004), HbA1c (P:0.011), TG (P:0.017) Compared to conservative treatment. 2. Adrenalectomy effectively reduced HbA1c after follow-up duration compared to the baseline of the surgical group. 3. There was no significant difference in the FBS (P:0.271), and overall lipids (P:0.421) control between Adrenalectomy and the Conservative group.
Salcuni (2016) (41)	32	21	Imp: 9 Wors: 0	5	Imp: 6 Wors: 1	Not reported	Mean BMI: 26.5 ± 4.0	–	–	23	14	Imp: 1 Wors: 11	9	Imp: 0 Wors: 8	Not reported	Mean BMI: 26.9 ± 4.2	–	–	1. Adrenalectomy was effective in improving HTN (P<0.0001), BS (P<0.0001) Compared to conservative treatment. 2. Adrenalectomy was NOT effective in improving HTN (P:0.44), DM (P:1), and BMI (P:0.98) after follow-up duration compared to the baseline of the surgical group.
Araujo-Castro)2022( (42)	42	Not reported	Change in SBP: −4.6 ± 17.47 and DBP: 1.16 ± 12.20	Not reported	Change in FBS: −16.6 ± 45.07 and HbA1c: −0.2 ± 0.75	Not reported	Change in BMI: 0.3 ± 2.83	Not reported	Change in HDL: 6.7 ± 20.38, LDL: −11.4 ± 37.44 and TG: −20.21 ± 55.97	217	Not reported	Change in SBP: −2.8 ± 22.35 and DBP: 0.1 ± 11.82	Not reported	Change in FBS: −1.0 ± 26.92 and HbA1c: −0.0 ± 1.01	Not reported	Change in BMI: 0.1 ± 3.05	Not reported	Change in HDL: 1.7 ± 10.01, LDL: −10.7 ± 34.56, and TG: 1.3 ± 59.23	1. Adrenalectomy was NOT effective in improving HTN, and BMI (P:0.54) after follow-up duration compared to the baseline of the surgical group. 2. Adrenalectomy was effective in improving FBS (P:0.035), TG (P:0.029) Compared to conservative treatment. 3. Adrenalectomy was effective in improving FBS (P:0.022), TG (P:0.024), HDL (P:0.039) after follow-up duration compared to the baseline of the surgical group. 4. Conservative treatment effectively reduced LDL (P:0.002) Compared to the baseline of the conservative group.
Petramala (2017) (6)	26	85% of MACS patients	58.82% of MACS patients	DM: 38% of MACS patients	DM: 35.5% of MACS patients	Obese: 53.8% of MACS patients	Obese: 24.5% of MACS patients	High TG:34% of MACS patients	High TG:27% of MACS patients	44	63.1% of MACS patients	72.5% of MACS patients	25% of MACS patients	38.5% of MACS patients	Obese: 33% of MACS patients	Obese: 42.7% of MACS patients	High TG:34% of MACS patients	High TG:38% of MACS patients	1. Adrenalectomy was effective in improving HTN (P<0.05), Obesity (P<0.05) Compared to conservative treatment. 2. Adrenalectomy was NOT effective in improving DM, overall lipid profile, Compared to conservative treatment.
Kawate (2014) (44)	15	10	Imp: 6 Wors: 1	5 DM or IGT	Imp: 3 Wors: 0	5	Not Reported	8	Imp: 1 Wors: 0	12	6	Imp: Wors: 9	5 DM or IGT	Imp: 1 Wors: 4	5	Not Reported	10	Imp: 1 Wors: 2	1. Adrenalectomy was effective in improving HTN, and DM after follow-up duration compared to the baseline of the surgical group. 2. Adrenalectomy was NOT effective in improving Dyslipidemia, after follow-up duration compared to the baseline of the surgical group.
Rossi (2000) (45)	5	4	Imp: 5 Wors: 0	3 DM	Imp: 3 Wors: 0	–	–	–	–	7	7	Imp: 0 Wors: 0	3 DM	Imp: 0 Wors: 0	–	–	–	–	1. Adrenalectomy was effective in improving HTN, and DM after follow-up duration compared to the baseline of the surgical group.
Liu (2020) (46)	31	22	Imp: 12 Wors: 0	9 IGT	Imp: 6 Wors: 0	11	Imp: 4 Wors: 0	Not Reported	Not Reported	11	8	Imp: 1 Wors: 0	4 IGT	Imp: 1 Wors: 0	6	Imp: 1 Wors: 0	Not Reported	Not Reported	1. Adrenalectomy effectively improved HTN, LDL, Total Cholesterol, and BMI after follow-up duration compared to the baseline of the surgical group. 2. Adrenalectomy was NOT effective in improving FBS, TG, after follow-up duration compared to the baseline of the surgical group.
Li (2017) (47)	64	46	Imp: 19 Wors: 0	13 DM	Imp: 7 Wors: 0	20	Imp: 25 Wors: 13	24	Imp: 5 Wors: 0	32	23	Imp: 1 Wors: 9	8 DM	Imp: 1 Wors: 8	9	Imp: 3 Wors: 13	10	Imp: 1 Wors: 6	1. Adrenalectomy effectively improved HTN after follow-up duration compared to the baseline of the surgical group. 2. Adrenalectomy was NOT effective in improving FBS, Dyslipidemia after follow-up duration compared to the baseline of the surgical group. 3. Adrenalectomy was effective in improving BW Compared to conservative treatment.
Ricciato (2014) (48)	16	Not Reported	Not Reported	Not Reported	Not Reported	–	–	Not Reported	Not Reported	17	Not Reported	Not Reported	Not Reported	Not Reported	–	–	Not Reported	Not Reported	1. Adrenalectomy effectively reduced SBP, DBP, FBS, HDL after follow-up duration compared to the baseline of the surgical group. 2. Conservative treatment was NOT effective in reducing SBP, Lipid profile but effective in reducing DBP compared to the baseline of the Conservative group.

Imp, Improvement; Wors, Worsen.

## 4 Discussion

Since the majority of patients with MACS are of an age range (in this study, the mean age for males was 56.1 years, and the mean age for females was 60.4 years) when HTN, diabetes, and obesity are highly prevalent (53–57), it is difficult to determine whether these metabolic complications are influenced by excess cortisol only or are affected by age either. However, some researches indicate that prolonged exposure to mild glucocorticoid excess resulting from adrenal incidentalomas is closely linked to a notable rise in cardiometabolic risk (22, 58). Both *in-vivo* and *in-vitro* evidence highlight how glucocorticoids (GCs) excess contribute to the pathophysiology of diabetes, osteoporosis, and dyslipidemia. Elevated cortisol levels affect blood glucose (on both liver and skeletal muscles), insulin sensitivity, and pancreatic function, linking MACS to a heightened risk of type 2 diabetes (59–67). Additionally, GCs excess adversely affects bone health by influencing osteoblast and osteocyte activity, leading to increased osteoclast activity and a potential risk of osteoporosis (68–71). In adipose tissue, GCs play a dual role in promoting both lipolysis and lipogenesis, contributing to dyslipidemia and adipose tissue changes as well as visceral obesity in conditions like MACS (60, 72–74). Additionally, it has been demonstrated that cortisol-mediated activation of the mineralocorticoid receptor may induce vascular changes even in mild and subclinical hypercortisolism (22). The outcomes might be associated with the severity and duration of hypersecretion and the sensitivity of each patient to cortisol (20). This systematic review and meta-analysis revealed that a notable proportion of patients with MACS experience various comorbidities. The findings of our study indicate that 62.9% of MACS patients have hypertension, 29.2% exhibit impaired glucose metabolism (DM or IGT), 41.4% suffer from dyslipidemia, and 38.2% are affected by obesity. Our findings are similar to the ENSAT NAPACA-Outcome Study conducted by Deutschbein et al., involving 3640 evaluated patients (7% with Autonomous Cortisol Secretion (ACS), 36% with possible Autonomous Cortisol Secretion (PACS), and 57% with NFA), the prevalence of cardiovascular risk factors among patients with PACS and ACS included higher rates of hypertension (72% and 73%, respectively), dyslipidemia (42% and 49%), and diabetes (22% and 25%). These rates were significantly higher compared to patients with NFA (25). [The 2016 ESE-ENSAT guideline introduced two categories for this condition at that time, defining patients as possible autonomous cortisol secretion (PACS) or autonomous cortisol secretion (ACS) based on post-1mg-DST cortisol levels (75)].

Based on the available and diverse published data, our analysis revealed a significant advantage of the adrenalectomy group compared to the conservative treatment group in general; However, it is crucial to consider that observational studies, by their design, cannot conclusively prove causality (76). Our meta-analysis revealed that SBP, DBP, and osteoporosis management of adrenalectomy were significantly better than conservative therapy. Additionally, in some included studies, the benefits of adrenalectomy in the control of dyslipidemia and obesity were reported but they were not significantly different in our subgroup analysis (28, 35, 39, 40, 42, 43, 46–48). It’s important to take into account that weight loss after abdominal surgery may influence parameters such as lipid profile and blood glucose levels (77, 78). thus, we cannot definitively attribute these outcomes solely to hormonal effects following adrenalectomy. Previous reviews revealed the same benefits of adrenalectomy in terms of cardiovascular risk factors (20, 79, 80). During the course of conservative treatment for MACS, it is possible for patients to experience a deterioration in their comorbidities, even when they are being appropriately monitored and receiving suitable medical therapy. However, reports almost indicated no worsening of hypertension and diabetes after surgical treatment. In our review, only two patients for hypertension and one patient for DM exhibited deterioration after surgical intervention.

The occurrence of adrenal insufficiency following adrenalectomy, even in cases of unilateral adrenalectomy (81–84), may lead to the hesitation in proceeding with the surgical procedure. Research findings indicate a significant decrease in post-operative stimulated cortisol levels in nearly 28% of the patients (85, 86). However, given that none of the included studies reported any incidence of adrenal insufficiency during the follow-up, this systematic review abstained from evaluating this particular aspect. moreover, Consideration must be given to surgical complications. Hemorrhage from the adrenal and renal veins or the adrenal cortex, injuries to the vena cava, puncture of the diaphragm, and laceration of the spleen represent the primary intraoperative complications. Postoperatively, prevalent complications include retroperitoneal hematoma, incisional hernia, pancreatic fistula, hyponatremia, and intestinal damage (87). Additionally, mortality has been documented in a limited number of adrenalectomy cases (88). BMI is one of the factors that contribute to increased complications after laparoscopic adrenalectomy (89, 90), and as we showed in this study, patients with MACS are at risk of obesity; therefore, the increase in the risk of laparoscopic adrenalectomy complications for these patients must be considered. Furthermore, patients with MACS are at older ages, which contributes to an increase in the risk of laparoscopic adrenalectomy complications (91, 92).

There is a lack of uniform and homogeneous standards for diagnosing and defining MACS in the included studies; as shown in **Table 2**, these variations in MACS definition could cause bias. All studies confirmed that there must not be any symptoms of overt Cushing’s syndrome, and, with the exception of one, all studies employed 1mg-DST, which previously has been shown to be the most sensitive screening test for an aberrant hypothalamic-pituitary-adrenal axis (93). However, the cortisol cut-off following DST differs across the included studies. The most recent guideline for managing adrenal incidentalomas, jointly developed by the European Society of Endocrinology and the European Network for the Study of Adrenal Tumors (ESE, ENSAT), recommends considering a 1-mg overnight dexamethasone suppression test with a cutoff value of (>1.8 µg/dL) to identify MACS (94). Previously, The National Institutes of Health (NIH), American Association of Clinical Endocrinologists (AACE), and American Association of Endocrine Surgeons (AAES) recommend >5.0 μg/dL, Endocrine Society (ES) and French Society of Endocrinology (FSE) recommend >1.8 μg/dL as a cut-off for DST (95).

It is unlikely that MACS can be considered an early stage of Cushing’s syndrome, as the progression of overt Cushing’s syndrome is rare among individuals with MACS. In our systematic review, only one female patient with MACS progressed to overt Cushing’s syndrome. Additional research has also demonstrated that the progression from MACS to Overt Cushing’s syndrome is infrequent (96–98). The ESE-ENSAT guideline also recommends against considering patients with MACS as being at high risk for the development of overt Cushing’s syndrome (94).

Previous systematic reviews which evaluated the effect of adrenalectomy on cardiovascular risk factors in patients with MACS showed the benefits of surgical treatment over conservative treatment, particularly regarding HTN, DM, and obesity (20, 79, 80). Furthermore, based on the ESE-ENSAT guideline, The recommendation for surgical intervention (Adrenalectomy) should be determined for all MACS patients as the standard care that aligns with and supports our findings (94).

## 5 Limitations

This systematic review and meta-analysis exhibit certain limitations. The definition and diagnostic criteria of MACS varied among the included studies, reflecting the absence of a universally accepted definition at that time. In our efforts to assess the improvement or worsening of comorbidities such as hypertension, diabetes mellitus, osteoporosis, dyslipidemia, and obesity, it is important to acknowledge that variations in the diagnostic criteria and definitions of the improvement or deterioration of each comorbidity among studies may impact our research outcomes. Another limitation of this study is the lack of clarity in the included studies regarding the specific details of conservative treatment. Consequently, we are unable to assess the pharmacological effects. Variations in conventional treatment, such as the type of medications used or dosages administered, could introduce discrepancies in the outcomes of conservative treatments across different studies, which indeed affect the accuracy. Furthermore, information regarding postoperative corticosteroid supplementation, such as the dosage, the quantity of patients who received corticosteroids, and the duration of drug administration, was not specified in the included studies. Nevertheless, it should be acknowledged as an important factor that may influence the outcomes of this study. The duration of follow-up is an additional important limitation that could notably impact the decision about adrenalectomy. It is not well recognized whether the durations of follow-ups were sufficient to determine the efficacy of each treatment. Also, the incomplete data in some studies made us to exclude them from our meta-analysis.

While our meta-analysis provides valuable insights, cautious interpretation is advised. The heterogeneity observed between studies, particularly in the sub-groups of BW, DBP, SBP, cholesterol, and TG (I2 > 30%), poses a significant limitation in the interpretability of these findings. This heterogeneity may arise from methodological differences, participant demographics, variations in the type of conservative treatment received by control groups across different studies, and other unaccounted factors. While this meta-analysis represents the only study conducted on this topic, future original trials with similar aims may either reinforce or weaken the findings of our study.

## 6 Conclusion

In this study, we showed that the presence of MACS is linked to an elevated likelihood of experiencing various Comorbidities. Additionally, this study demonstrated the advantage of adrenalectomy over conservative treatment for MACS-related comorbidities. However, heterogeneous data were used in current research. Additionally, the patients undergoing adrenalectomy experienced an overall improvement in cardiovascular risk factors compared to their initial baseline characteristics. However, in patients receiving conservative care, cardiovascular risk factors may deteriorate. Further and more accurate research, particularly in terms of follow-up duration and sample size, is required to make a precise decision.

## Data availability statement

The original contributions presented in the study are included in the article/**Supplementary Material**, further inquiries can be directed to the corresponding authors.

## Author contributions

MMK: Conceptualization, Data curation, Investigation, Supervision, Writing – original draft, Writing – review & editing. SM: Methodology, Writing – review & editing. HH: Formal analysis, Methodology, Software, Writing – original draft. MPS: Conceptualization, Resources, Writing – review & editing. MAK: Writing – original draft, Writing – review & editing. RA: Methodology, Writing – review & editing. SS: Formal analysis, Methodology, Writing – review & editing. MM-T: Resources, Supervision, Writing – review & editing. BL: Methodology, Supervision, Writing – review & editing.
