# Supplementary material for: Comparison of adrenalectomy with conservative treatment on mild autonomous cortisol secretion: a systematic review and meta-analysis
Source: Front Endocrinol (Lausanne). 2024 May 13;15:1374711. doi: 10.3389/fendo.2024.1374711 (PMC11131104; doi:10.3389/fendo.2024.1374711)
Supplement: Supplementary file 1 [file DataSheet_1.docx]

**Surgical & conservative management & Cushing syndrome**

**Search date: 20 December 2023**

**Pubmed:**

(surg*[TIAB] OR operative[TIAB] OR (invasive[TIAB] AND procedures[TIAB] ) OR operations[TIAB] OR transplantation*[TIAB] OR "Surgical Procedures, Operative"[Mesh] OR "General Surgery"[Mesh] OR "Adrenalectomy"[Mesh] OR Adrenalectom*[TIAB] OR ((Excision[TIAB] OR Remov*[TIAB] OR Resection[TIAB] ) AND (adrenal[TIAB] AND gland*[TIAB])) )

**AND**

("Conservative Treatment"[Mesh] OR ( Conservative[TIAB] AND (Treatment*[TIAB] OR Therap*[TIAB] OR management*[TIAB] OR Cure[TIAB] ) ) )

**AND**

("Cushing Syndrome"[Mesh] OR Hypercortisolism[TIAB] OR (Inappropriate[TIAB] AND ACTH[TIAB] AND Secretion[TIAB] ) OR (Inappropriate [TIAB] AND Adrenocorticotropic[TIAB] AND Secretion[TIAB] ) OR cushing*[TIAB] OR Hypercortisolism[TIAB] OR (excess [TIAB] AND (level[TIAB] OR levels[TIAB] ) AND cortisol[TIAB] ) OR Hydrocortisone[TIAB] OR Glucocorticoids[TIAB] OR (mild [TIAB] AND autonomous[TIAB] AND cortisol[TIAB] AND secretion[TIAB] ) OR MACS [TIAB] )

**RESULTS: 152**

**WEB OF SCIENCE:**

**TS**=((surg* OR operative OR (invasive AND procedures ) OR operations OR transplantation* OR Adrenalectom* OR ((Excision OR Remov* OR Resection ) AND (adrenal AND gland*)) )

**AND**

( Conservative AND (Treatment* OR Therap* OR management* OR Cure ) )

**AND**

( Hypercortisolism OR (Inappropriate AND ACTH AND Secretion ) OR (Inappropriate AND Adrenocorticotropic AND Secretion ) OR cushing* OR Hypercortisolism OR

(excess AND (level OR levels ) AND cortisol )

OR Hydrocortisone OR Glucocorticoids OR (mild AND autonomous AND cortisol AND secretion ) OR MACS ))

**RESULTS= 172**

**SCOPUS:**

TITLE-ABS-KEY ( (surg* OR operative OR (invasive AND procedures ) OR operations OR transplantation* OR Adrenalectom* OR ((Excision OR Remov* OR Resection ) AND (adrenal AND gland*)) )

**AND**

( Conservative AND (Treatment* OR Therap* OR management* OR Cure ) )

**AND**

( Hypercortisolism OR (Inappropriate AND ACTH AND Secretion ) OR (Inappropriate AND Adrenocorticotropic AND Secretion ) OR cushing* OR Hypercortisolism OR

(excess AND (level OR levels ) AND cortisol )

OR Hydrocortisone OR Glucocorticoids OR (mild AND autonomous AND cortisol AND secretion ) OR MACS ))

**RESULTS: 1012**

**EMBASE:**

**('surgery'/exp OR 'general surgery'/exp OR** surg*:ti,ab,kw OR operative:ti,ab,kw OR (invasive:ti,ab,kw AND procedures:ti,ab,kw ) OR operations:ti,ab,kw OR transplantation*:ti,ab,kw OR 'adrenalectomy'/exp OR Adrenalectom*:ti,ab,kw OR ((Excision:ti,ab,kw OR Remov*:ti,ab,kw OR Resection:ti,ab,kw ) AND (adrenal:ti,ab,kw AND gland*:ti,ab,kw )) )

**AND**

**('conservative treatment'/exp OR (** Conservative:ti,ab,kw AND (Treatment*:ti,ab,kw OR Therap*:ti,ab,kw OR management*:ti,ab,kw OR Cure:ti,ab,kw ) ) )

**AND**

('Cushing syndrome'/exp OR Hypercortisolism:ti,ab,kw OR (Inappropriate:ti,ab,kw AND ACTH:ti,ab,kw AND Secretion:ti,ab,kw ) OR (Inappropriate:ti,ab,kw AND Adrenocorticotropic:ti,ab,kw AND Secretion:ti,ab,kw ) OR cushing*:ti,ab,kw OR Hypercortisolism:ti,ab,kw OR

(excess:ti,ab,kw AND (level:ti,ab,kw OR levels:ti,ab,kw ) AND cortisol:ti,ab,kw )

OR Hydrocortisone:ti,ab,kw OR Glucocorticoids:ti,ab,kw OR (mild:ti,ab,kw AND autonomous:ti,ab,kw AND cortisol:ti,ab,kw AND secretion:ti,ab,kw ) OR MACS:ti,ab,kw )

**Results: 1157**

Reports excluded:

Reason 1 (n = )

Reason 2 (n = )

Reason 3 (n = )

etc.

Reports not retrieved

(n = )
