# Supplementary figures and images for: Comparison of adrenalectomy with conservative treatment on mild autonomous cortisol secretion: a systematic review and meta-analysis
Source: Front Endocrinol (Lausanne). 2024 May 13;15:1374711. doi: 10.3389/fendo.2024.1374711 (PMC11131104; doi:10.3389/fendo.2024.1374711)

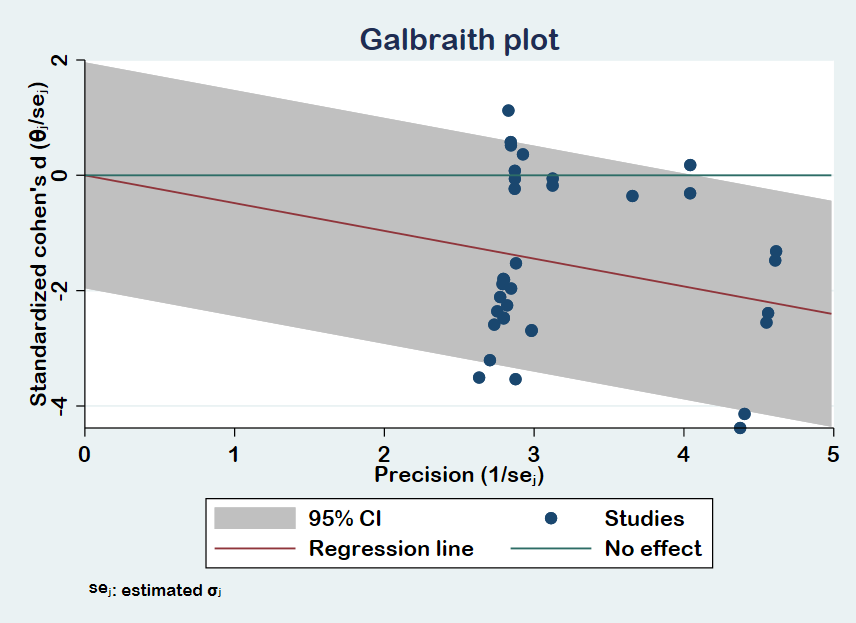

Supplement: Supplementary file 2 [file Image_1.tif]

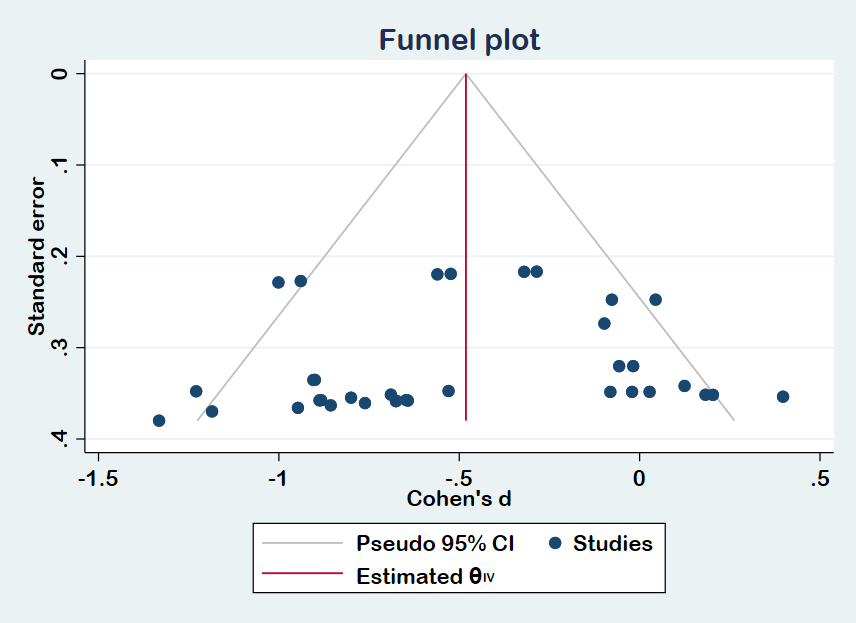

Supplement: Supplementary file 3 [file Image_2.tif]

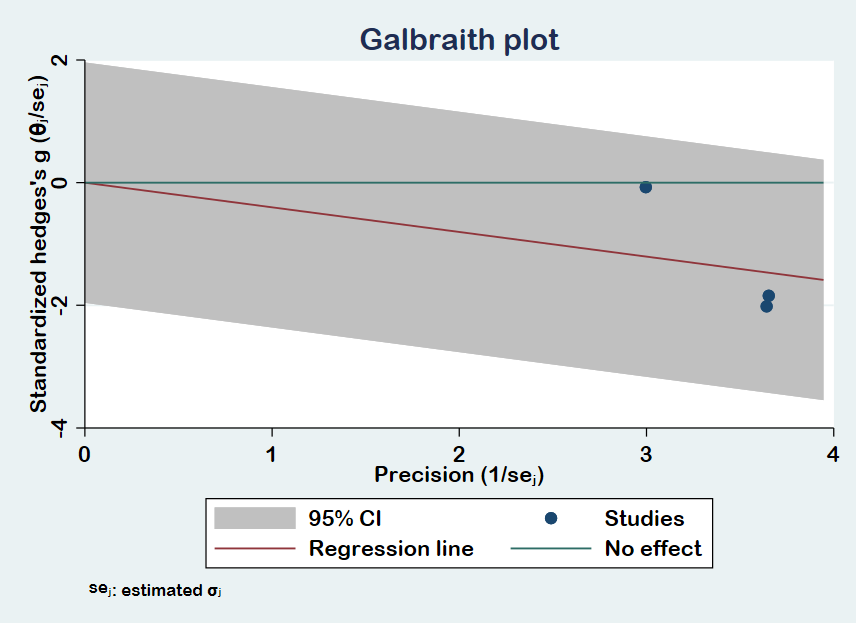

Supplement: Supplementary file 4 [file Image_3.tif]

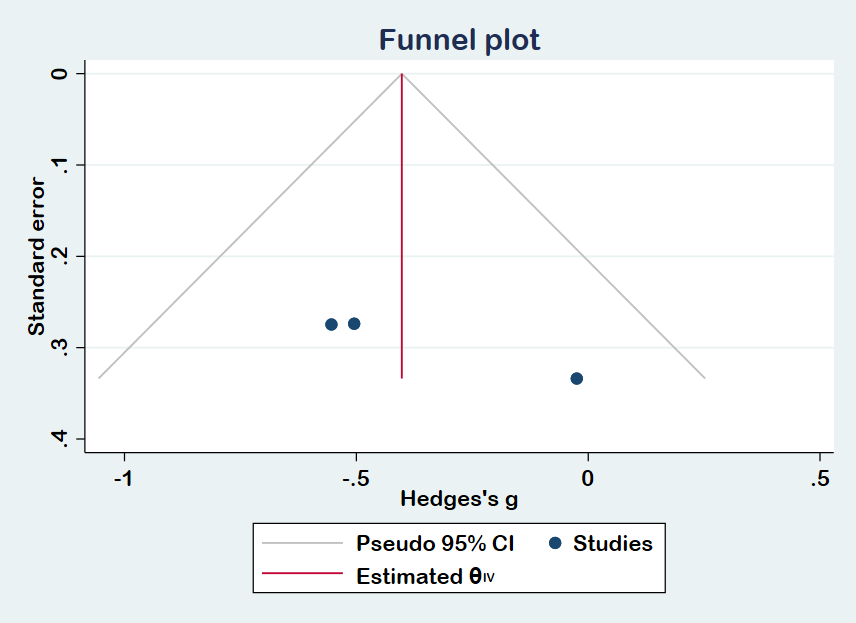

Supplement: Supplementary file 5 [file Image_4.tif]

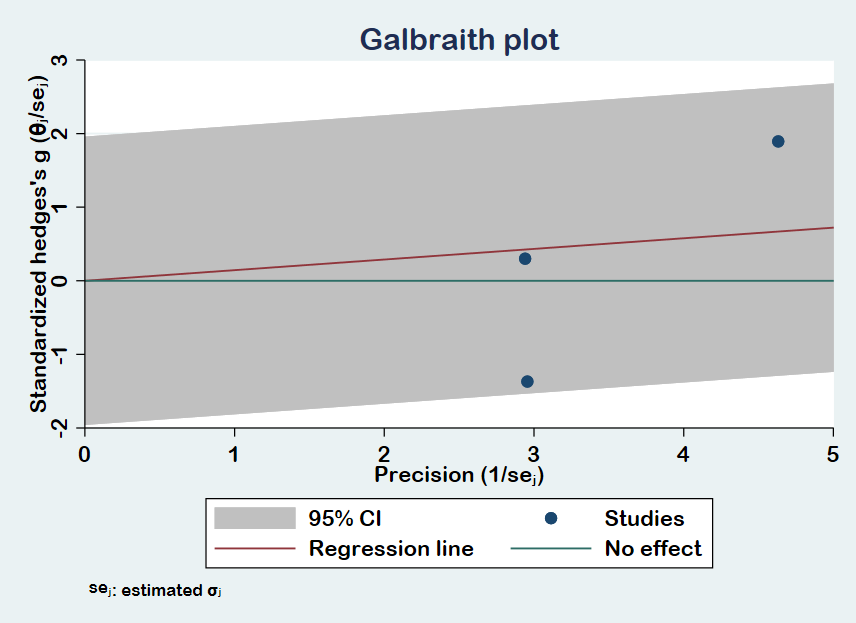

Supplement: Supplementary file 6 [file Image_5.tif]

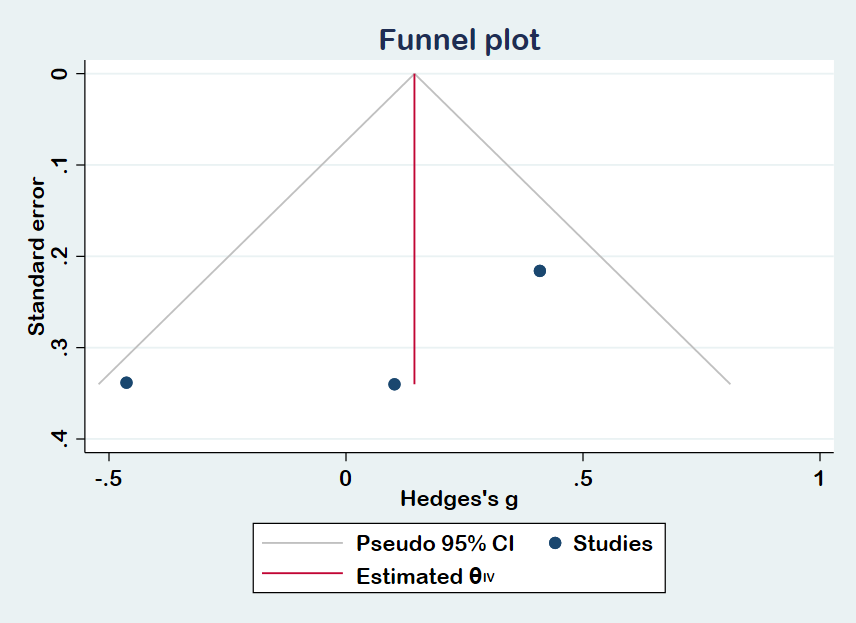

Supplement: Supplementary file 7 [file Image_6.tif]
